# SARS-CoV-2 Humoral Immunity Persists Following Rituximab Therapy

**DOI:** 10.3390/vaccines11121864

**Published:** 2023-12-18

**Authors:** Liangjian Lu, Chang Yien Chan, Yi Yang Lim, Mya Than, Sharon Teo, Perry Y. W. Lau, Kar Hui Ng, Hui Kim Yap

**Affiliations:** 1 Department of Paediatrics, Khoo Teck Puat - National University Children’s Medical Institute, National University Health System, Singapore 119228, Singaporesharon_teo@nuhs.edu.sg (S.T.); paenkh@nus.edu.sg (K.H.N.); paeyaphk@nus.edu.sg (H.K.Y.); 2 Department of Paediatrics, Yong Loo Lin School of Medicine, National University of Singapore, Singapore 117549, Singapore; paeumt@nus.edu.sg

**Keywords:** SARS-CoV-2, mRNA vaccines, long-lived plasma cells, Rituximab

## Abstract

Long-term humoral immunity is mediated by short-lived plasma cells (replenished by memory B cells) and long-lived plasma cells. Their relative contributions are uncertain for immunity to SARS-CoV-2, especially given the widespread use of novel mRNA vaccines. Yet, this has far-reaching implications in terms of the need for regular booster doses in the general population and perhaps even revaccination in patients receiving B cell-depleting therapy. We aimed to characterise anti-SARS-CoV-2 antibody titres in patients receiving Rituximab following previous SARS-CoV-2 vaccination. We recruited 10 fully vaccinated patients (age: 16.9 ± 2.52 years) with childhood-onset nephrotic syndrome, not in relapse, receiving Rituximab for their steroid/calcineurin-inhibitor sparing effect. Antibodies to SARS-CoV-2 spike (S) and nucleocapsid (N) proteins were measured immediately prior to Rituximab and again ~6 months later, using the Roche Elecys^®^ Anti-SARS-CoV-2 (S) assay. All ten patients were positive for anti-S antibodies prior to Rituximab, with six patients (60%) having titres above the upper limit of detection (>12,500 U/mL). Following Rituximab therapy, there was a reduction in anti-S titres (*p* = 0.043), but all patients remained positive for anti-S antibodies, with five patients (50%) continuing to have titres >12,500 U/mL. Six patients (60%) were positive for anti-N antibodies prior to Rituximab. Following Rituximab therapy, only three of these six patients remained positive for anti-N antibodies (*p* = 0.036 compared to anti-S seroreversion). Humoral immunity to SARS-CoV-2 is likely to be mediated in part by long-lived plasma cells.

## 1. Introduction

The widespread adoption of vaccines against SARS-CoV-2, in particular the Pfizer-BioNTech BNT162b2 and Moderna mRNA-1273 mRNA vaccines, which were approved in December 2020, has contributed to controlling mortality rates and facilitated a relaxation of pandemic-era public health measures [1]. However, as early as October 2021, there were concerns about the waning efficacy of mRNA vaccines in preventing symptomatic disease [2] associated with falling anti-spike antibody titres [3]. Since then, serial mRNA vaccine booster doses have received emergency use authorisation almost on an annual basis, albeit partially in response to the emergence of novel SARS-CoV-2 strains. Taking BNT162b2, for instance, a first and second booster dose were authorised in August 2021 and March 2022, respectively [4], followed by a bivalent dose encoding Omicron BA.4/BA.5 in addition to the ancestral SARS-CoV-2 strain in August 2022, and most recently a 2023–2024 formulation tailored against Omicron XBB.1.5 in September 2023 [5]. The optimal vaccination strategy going forward remains uncertain. While antigenic drift provides one rationale for subsequent booster doses, an equally important consideration is whether current vaccines against SARS-CoV-2 are able to produce long-lasting immunity. Failure to do so may mean that medically vulnerable patients will require regular booster doses, regardless of the degree of antigenic drift. 

Several vaccines in common use are known to produce long-lasting humoral immunity, with the half-lives of antibodies against rubella, mumps, and measles estimated at 114, 542, and 3014 years, respectively [6]. In contrast, some vaccines, such as seasonal influenza and RTS,S malaria vaccines, only lead to short-lived humoral responses lasting less than one year [7]. While the initial antibody response post-vaccination is mediated by plasmablasts and short-lived plasma cells, the ability of vaccines to induce long-lived plasma cells (LLPC) is key to producing long-lasting humoral immunity [7,8].

It is currently uncertain whether vaccines against SARS-CoV-2, in particular mRNA vaccines, are able to induce long-lived plasma cells and persistent humoral immunity. A recent study [9] was able to detect spike-specific plasma cells in bone marrow samples from fully vaccinated individuals, but only 10% of spike-specific plasma cells had a long-lived phenotype (CD19−CD45−) compared to 25% of all plasma cells. Moreover, while it has been argued that the rapid decline of anti-spike titres is inconsistent with LLPC induction [8], almost all antibody titres following vaccination will initially decline due to apoptosis of plasmablasts and short-lived plasma cells but may subsequently stabilise at levels adequate for protection, as has been demonstrated for the human papillomavirus vaccine [7,10]. 

Apart from careful longitudinal follow-up, the clinical use of CD20-depleting agents such as Rituximab provides a natural experiment that enables us to assess the contribution of long-lived plasma cells to SARS-CoV-2 humoral immunity. Rituximab is a chimeric monoclonal anti-CD20 antibody originally developed for the therapy of B cell malignancies and is currently incorporated into the standard of care for several B cell lymphomas [11,12]. It is also increasingly utilised in a wide variety of autoimmune disorders, ranging from pemphigus to neuromyelitis optica [11], and has more recently been adopted in difficult idiopathic nephrotic syndrome [12,13,14].

Rituximab efficiently depletes B cells through a combination of complement-dependent cytotoxicity and antibody-dependent cellular toxicity, as well as a direct effect on B cells driving apoptosis [11]. However, importantly, plasma cells are spared. This is because plasma cells do not express CD20, unlike all other B cells, which will be efficiently depleted by Rituximab therapy for about 6 months [11,15]. This implies that serological titres 6 months post-Rituximab will reflect the contribution of LLPCs given that the half-life of IgG is at best 23 days, with the remaining isotypes having shorter half-lives. Hence, we aimed to compare titres against SARS-CoV-2 spike (S) and nucleocapsid (N) protein in fully vaccinated individuals before and 6 months following Rituximab therapy in a cohort of patients with childhood-onset idiopathic nephrotic syndrome.

## 2. Methods

### 2.1. Patient Recruitment

Fully vaccinated patients with childhood-onset idiopathic nephrotic syndrome who were scheduled to receive Rituximab for clinical indications between June 2022 and January 2023 were recruited. Vaccination with mRNA vaccines in Singapore took place under a national vaccination programme, which began for our patients 12 years old and older in June 2021 and for patients between 5 and 11 years old in December 2021. In keeping with prevailing recommendations by the Singapore Expert Committee on COVID-19 vaccination (EC19V) at the time of study recruitment, fully vaccinated referred to having received primary vaccination comprising two doses of mRNA vaccines, as well as a booster dose typically 5 months after, but not later than 270 days from, the last primary vaccination dose [16,17]. In addition, patients also had the option to receive (i) a third additional primary vaccination dose due to their immunocompromised status in discussion with their physicians since September 2021 [18] and (ii) a further booster dose 5 months (subsequently one year) following their previous booster dose since October 2022 [19,20,21]. Indications for Rituximab therapy included a failure of mycophenolate mofetil or calcineurin inhibitors (CNIs) to produce an adequate steroid-sparing effect or CNI toxicity. Patients were excluded if they were in a nephrotic relapse. Blood samples were obtained just before the Rituximab infusion, as well as approximately 6 months after Rituximab. Informed consent/assent was obtained from all subjects and/or their parents. Ethical approval was obtained from the National Healthcare Group Domain Specific Review Boards (2008/00068) and conducted in accordance with the approved guidelines and regulations.

### 2.2. Laboratory Analyses

Titres against SARS-CoV-2 nucleocapsid and spike proteins were measured in the NUH Referral Laboratories using the Elecys^®^ Anti-SARS-CoV-2 (Roche, Basel, Switzerland) and anti-SARS-CoV-2 S assays (Roche, Basel, Switzerland), respectively. The anti-SARS-CoV-2 assay is a qualitative assay, while the anti-SARS-CoV-2 S assay has a detection range of 0.40 to 12,500 U/mL, and a concentration of >0.80 U/mL is defined as positive for SARS-CoV-2 spike antibody per the manufacturer’s instructions [22].

B cell subsets were identified using the following mouse anti-human monoclonal antibodies (Becton Dickinson, Franklin Lakes, NJ, USA): CD19 (SJ25C1), CD27 (M-T271), CD45 (2D1), and IgD (IA6-2). Whole blood was washed three times with 1× phosphate-buffered saline (1× PBS) before staining. Antibody staining was conducted using 100 µL of washed whole blood for 15 min at room temperature. Red blood cells were lysed with FACS Lysing Solution (Becton Dickinson, Franklin Lakes, NJ, USA) for 10 min at room temperature. Cells were then washed twice with 1× PBS and re-suspended in 1× PBS for acquisition through flow cytometry (FACSCanto II, Becton Dickinson). Data analysis for the determination of the percentage of positive cells was performed using FlowJo (Becton Dickinson). Switched memory B cells were defined as CD27+IgD− subset in the total B cell (CD19+) populations.

### 2.3. Statistical Analysis

Averages are given as means ± standard error unless otherwise stated. The antibody half-life was calculated assuming exponential decay. Anti-spike titres before and after Rituximab were compared using the Wilcoxon signed rank test (modelling titres > 12,500 U/mL as 12,500 U/mL), while other paired comparisons were performed using paired *t*-tests. The different rates of seroreversion for anti-spike and anti-nucleocapsid antibodies were compared using Fisher’s exact test. *p* ≤ 0.05 was taken as the threshold of statistical significance. Statistical analyses were performed using Microsoft Excel version 2111 (Microsoft, Redmond, WA, USA) and SPSS version 21 (IBM, Armonk, NY, USA).

## 3. Results

### 3.1. Patient Characteristics

**Figure 1:**
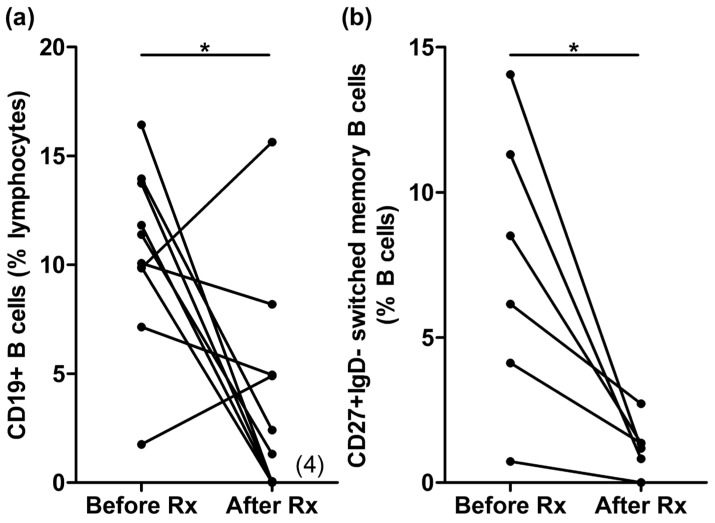
B cell depletion following Rituximab (Rx) therapy. (**a**) Total B cells (CD19+) and (**b**) switched memory B cells (CD27+IgD−) before and ~6 months following Rituximab. The numerals in brackets denote the number of patients with (near) undetectable levels of total B cells. * refers to *p* < 0.05.

Ten patients were enrolled in the study, with cohort characteristics as shown in Table 1. Patients were well vaccinated, with nine (90%) receiving at least three vaccine doses, with the most recent dose being 4.6 ± 1.11 months prior to Rituximab. B cells were depleted following Rituximab, as even at 6 months, the CD19 population was still decreased at 3.8 ± 1.59% compared to 10.6 ± 1.29% prior to Rituximab (*p* = 0.018) (Figure 1a). Quantified in absolute terms, this corresponded to a decrease in the CD19 population from 272 ± 40 cells/µL before Rituximab to 116 ± 61 cells/µL after Rituximab (*p* = 0.024) (Figure S1). Specifically, four patients (40%) still had virtually undetectable B cells at six months, i.e., <0.1% total lymphocytes. Even in the remaining six patients with detectable B cells, only a small proportion of these B cells were class-switched memory B cells, i.e., 1.24 ± 0.362% compared to 7.48 ± 1.98% prior to Rituximab (*p* = 0.024) (Figure 1b), suggesting that the detectable B cells were driven by B cell repopulation.

### 3.2. Anti-Spike Antibodies

**Figure 2:**
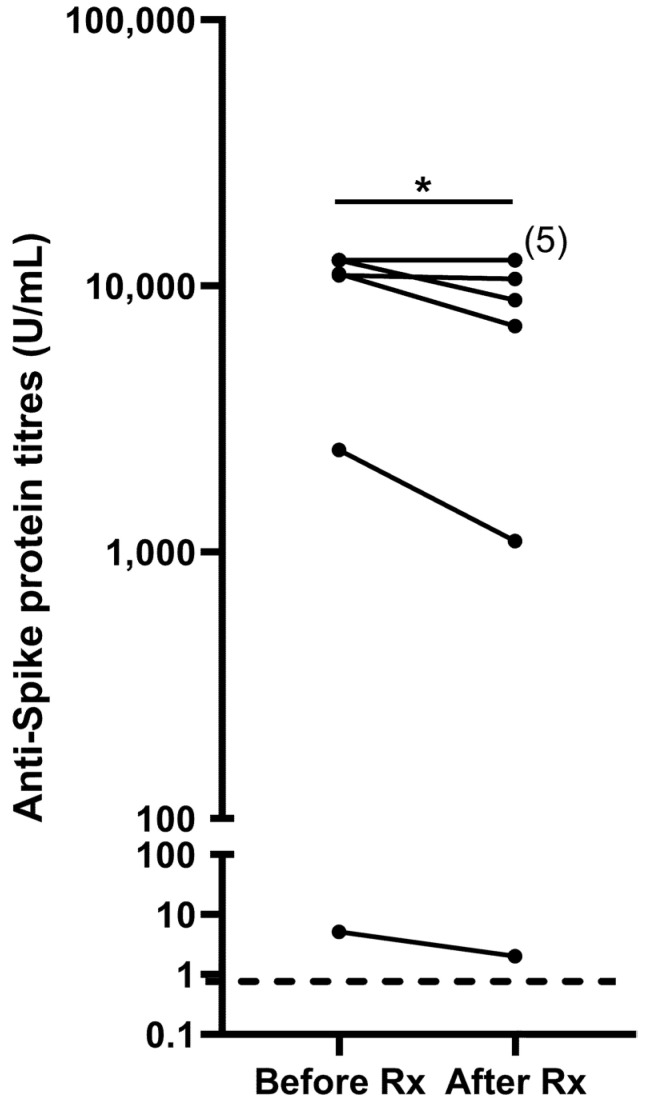
Anti-spike antibodies before and ~6 months following Rituximab (Rx). Six patients had baseline anti-spike protein titres > 12,500 U/mL, which are reflected as 12,500 U/mL. The numerals in brackets denote the number of patients continued to maintain titres > 12,500 U/mL after Rituximab. The dotted line denotes the threshold for anti-spike protein titre positivity, i.e., 0.8 U/mL. * refers to *p* < 0.05.

All 10 patients were seropositive for anti-spike antibodies prior to Rituximab, with 6 patients (60%) having levels above the upper limit of detection, i.e., >12,500 U/mL. Following Rituximab, there was a decrease in anti-spike titres (*p* = 0.043, Figure 2). However, this decrease was small in magnitude. All patients continued to be seropositive, and 5 patients continued to have anti-spike titres > 12,500 U/mL. Moreover, of the 4 patients with quantifiable anti-spike titres at baseline, the decrease in titres post-Rituximab was 39 ± 12.9%, corresponding to an antibody half-life of 9 months (95% confidence interval: 4–31 months).

### 3.3. Anti-Nucleocapsid Antibodies

Six patients (60%) were seropositive for anti-nucleocapsid antibodies prior to Rituximab. Of these six patients, only three remained seropositive following Rituximab. This corresponded to a 50% rate of sero-reversion, which was higher than that for anti-spike antibodies at 0% (*p* = 0.036). One seronegative patient became seropositive during the course of the study. This was associated with clinically asymptomatic infection, and anti-spike antibody titres remained >12,500 U/mL before and following Rituximab.

## 4. Discussion

Using a small paediatric cohort treated with Rituximab, we demonstrate that anti-spike antibodies following vaccination remain robust even after B cell depletion, in keeping with the induction of long-lived plasma cells. Similarly, while anti-nucleocapsid antibodies displayed a higher sero-reversion rate of 50% at 6 months, this is similar to that reported in predominantly healthy cohorts comprising healthcare workers [23,24]. These findings are novel, as while there are numerous studies investigating the effect of *prior* Rituximab therapy on SARS-CoV-2 vaccine immunogenicity [25,26], there have been minimal efforts so far to characterise the effect of Rituximab therapy *following* SARS-CoV-2 vaccination on an established immunological response [27]. Indeed, to the best of our knowledge, this has only been specifically studied recently in a single study involving 31 adult patients with a variety of autoimmune conditions, including ANCA-associated vasculitis, rheumatoid arthritis, and membranous glomerulonephritis [27]. Similar to our findings, although anti-spike antibodies declined over 6 months, 96% of participants remained seropositive at the end of the study. However, significant limitations of that study include 29% of patients receiving a booster vaccine dose during the study period, incomplete follow-up (20% dropout), a lack of data on CD19 counts as well as anti-nucleocapsid titres, concerns that are addressed, at least in part, by our study.

In addition, beyond demonstrating the robustness of anti-spike titres to Rituximab therapy, it is also significant that the estimated anti-spike antibody t_1/2_ of 9 months in our cohort is somewhat longer than the estimate of 52 days (95% confidence interval: 46–58) obtained from participants receiving two doses of mRNA-1273 [28]. Indeed, besides the larger total number of vaccine doses received, our estimate should be better interpreted as reflecting hybrid immunity, given the high prevalence of anti-nucleocapsid antibodies in our cohort, which has been associated with longer-lasting humoral responses to and clinical protection from SARS-CoV-2 infection [8,29,30,31]. 

The potential importance of hybrid immunity in generating durable humoral immunity is further highlighted by two observations in this cohort. First, hybrid immunity may explain the lower sero-reversion rate of anti-spike antibodies compared to anti-nucleocapsid antibodies. This is because while SARS-CoV-2 infections expose subjects to both spike and nucleocapsid antigens, most vaccines against SARS-CoV-2, in particular mRNA vaccines, encode only the spike protein. Hence, the repeated challenge with the spike protein during booster vaccination has enabled the development of a long-lived anamnestic response to the spike protein, in contrast to a shorter-lived response to the nucleocapsid protein, from which antigenic exposure only occurs during infection alone [32]. Moreover, due to the different routes of administration, SARS-CoV-2 vaccines may be better able to induce a systemic humoral response compared to wild-type infection alone, which predominantly resides in the respiratory tract, although, interestingly, the latter may be better able to induce mucosal immunity, which could be a closer correlate of protection [33,34,35,36]. 

Second, we have previously shown in a paediatric cohort of COVID-naive patients that vaccine responses, including both humoral and T-cell responses, are markedly attenuated in immunocompromised patients compared to controls, including patients with idiopathic nephrotic syndrome [22]. The fact that anti-spike titres appear to be longer lived in this immunocompromised cohort compared to largely COVID-naïve cohorts from which initial anti-spike antibody t_1/2_ data are derived showcases the importance of hybrid immunity in producing long-lived humoral immunity, presumably by LLPC induction. 

The induction of LLPCs is in keeping with emerging data from longitudinal follow-up of large cohorts following vaccination, which suggests a declining rate of decay of antibodies [3,37,38] that may precede the eventual development of a stable plateau driven by LLPC antibody production. Indeed, in a large study (n = 8395), spike-specific antibodies seemed to decline following single exponential decay [38,39]. Nevertheless, the level at which the titre plateaus remains uncertain, and the clinical correlates of this titre are also still unknown. Yet, it may still be of clinical utility, given the change in focus from sterilising immunity, which requires high levels of neutralising antibodies, to protection from severe disease [40].

The limitations of this study include the small cohort size limited to a single disease, the heterogenous history of previous infections, and the lack of measurement of neutralising antibodies. Hence, this study will benefit from future work involving a larger number of Rituximab-treated patients across more diseases, which will also allow any heterogeneity in patient characteristics to be systemically adjusted for. Nevertheless, given the common mechanism of Rituximab in many autoimmune conditions, i.e., therapeutic B cell depletion, these findings may be reassuring to clinicians treating patients with Rituximab in other conditions beyond nephrotic syndrome. In addition, this study also showcases the utility of patient cohorts with Rituximab in identifying the source of antibodies, i.e., plasmablasts/short-lived plasma cells replenished by B cells vs. long-lived plasma cells. Similar data have been used by others to argue that tetanus and pneumococcal vaccines induce long-lived plasma cells [7,41] and even to deduce the source of autoantibodies in various autoimmune conditions such as pemphigus and systemic lupus erythematosus [15].

## 5. Conclusions

In conclusion, in a paediatric cohort with childhood-onset nephrotic syndrome treated with Rituximab, we show that anti-spike antibodies are relatively preserved following treatment with B cell-depleting agents. This is in keeping with the induction of long-lived plasma cells and suggests an element of durable immunity, which may reduce the need for frequent annual booster vaccinations in the general population. This has important implications given the far-reaching implications of any SARS-CoV-2 vaccine recommendations, including exposing the population to the risk of vaccine-related adverse effects as well as further increasing direct and indirect healthcare costs.

## Figures and Tables

**Table 1:** Cohort characteristics.

Characteristics	n = 10
Age at Rituximab (years)	16.9 ± 2.52
Female (%)	3 (30)
Number of Rituximab doses (%) - One - Two	7 (70) 3 (30)
Number of SARS-CoV-2 vaccine doses received (%) - Two - Three - Four	1 (10) 6 (60) 3 (30)
Immunosuppression at baseline before Rituximab - Corticosteroids - Mycophenolate mofetil - Calcineurin inhibitors	3 (30) 8 (80) 7 (70)
Interval between last vaccine dose and first Rituximab dose (months)	4.6 ± 1.11
Interval between first Rituximab dose and post-Rituximab blood sample (months)	6.6 ± 0.15
CD19+ B cells before Rituximab (% lymphocytes)	10.6 ± 1.29
CD19+ B cells after Rituximab (% lymphocytes)	3.8 ± 1.59
Switched memory B cells before Rituximab (% B cells)	12.9 ± 3.69
Switched memory B cells after Rituximab (% B cells)	1.24 ± 0.362

## Data Availability

Data are contained within the article and Supplementary Materials.

## Supplementary Materials

The following supporting information can be downloaded at: https://www.mdpi.com/article/10.3390/vaccines11121864/s1, Figure S1: Total B cells (CD19+) counts before and ~6 months following Rituximab.
