# Supplementary material for: SARS-CoV-2 Humoral Immunity Persists Following Rituximab Therapy
Source: Vaccines (Basel). 2023 Dec 18;11(12):1864. doi: 10.3390/vaccines11121864 (PMC10748262; doi:10.3390/vaccines11121864)
Supplement: Supplementary file 1 [file vaccines-11-01864-s001.zip › vaccines-2727440-supplementary.pdf]

vaccines

MDPI

Supplementary Materials

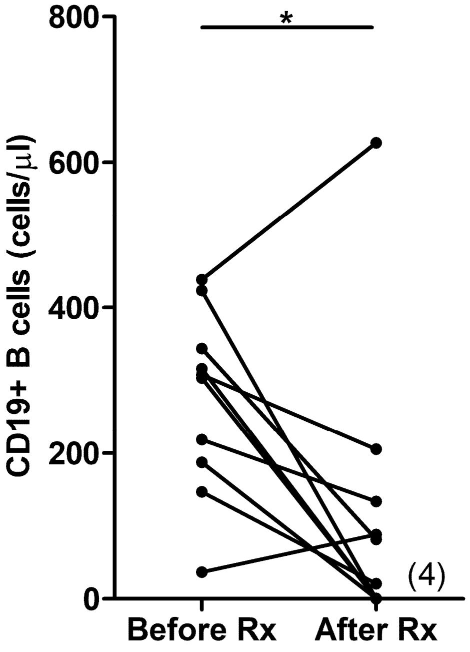

Figure S1: Total B cells (CD19+) counts before and ~6 months following Rituximab. The numerals in brackets denote the number of patients with (near) undetectable levels of total B cells. * refers to p< 0.05.
